# Review: *Veratrum californicum* Alkaloids

**DOI:** 10.3390/molecules26195934

**Published:** 2021-09-30

**Authors:** Madison L. Dirks, Jared T. Seale, Joseph M. Collins, Owen M. McDougal

**Affiliations:** 1 Department of Chemistry and Biochemistry, Boise State University, Boise, ID 83725, USA; madisondirks@u.boisestate.edu (M.L.D.); jaredseale@u.boisestate.edu (J.T.S.); 2 Biomolecular Sciences Ph.D. Program, Boise State University, Boise, ID 83725, USA; josephcollins177@u.boisestate.edu

**Keywords:** *Veratrum californicum*, alkaloid, drug discovery, cyclopamine, natural products

## Abstract

*Veratrum* spp. grow throughout the world and are especially prevalent in high mountain meadows of North America. All parts of *Veratrum* plants have been used for the treatment of ailments including injuries, hypertension, and rheumatic pain since as far back as the 1600s. Of the 17–45 *Veratrum* spp., *Veratrum californicum* alkaloids have been proven to possess favorable medicinal properties associated with inhibition of hedgehog (Hh) pathway signaling. Aberrant Hh signaling leads to proliferation of over 20 cancers, including basal cell carcinoma, prostate and colon among others. Six of the most well-studied *V. californicum* alkaloids are cyclopamine (**1**), veratramine (**2**), isorubijervine (**3**), muldamine (**4**), cycloposine (**5**), and veratrosine (**6**). Recent inspection of the ethanolic extract from *V. californicum* root and rhizome via liquid chromatography–mass spectrometry has detected up to five additional alkaloids that are proposed to be verazine (**7**)**,** etioline (**8**), tetrahydrojervine (**9**)**,** dihydrojervine (**10**), 22-keto-26-aminocholesterol (**11**). For each alkaloid identified or proposed in *V. californicum*, this review surveys literature precedents for extraction methods, isolation, identification, characterization and bioactivity to guide natural product drug discovery associated with this medicinal plant.

## 1. Introduction

**Figure 1:**
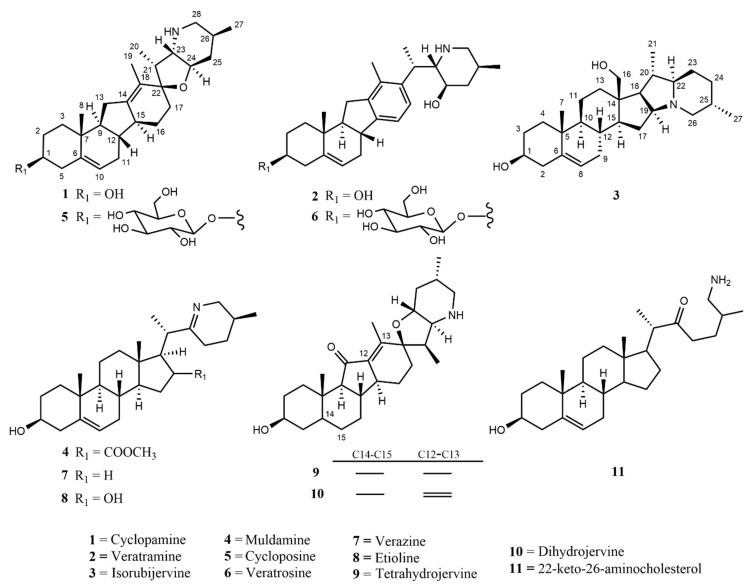
Structures of 11 known and proposed alkaloids in *Veratrum californicum*.

The genus *Veratrum* consists of 17–45 spp., most of which naturally occur in Asia, and all of them located exclusively in the Northern hemisphere. These perennials can either be classified as part of the Liliaceae or the Melanthiaceae family [1]. Typically, the species of *Veratrum* have been classified by their gross morphology, but the wide variety within the species has led to an absence of defined taxonomy [2]. In 2003, Zomlefer et al. classified *Veratrum* spp. by examination of their nuclear ribosomal internal transcribed spacers (ITS) [3]. This ITS method was correlated to traditional taxonomic classification by corresponding analysis of flower color and geographical location [3]. Table 1 lists the geographic region, a corresponding flower image, and the alkaloids and other relevant active components for representative *Veratrum* spp. that are pertinent to the current review. Based on alkaloid composition, the *Veratrum* spp. that will be discussed in this review are *V. lobelianum*, *V. grandiflorum*, *V. oxysepalum*, *V. maackii*, *V. nigrum*, *V. taliense*, *V. viride*, *V. eschscholtzii* and *V. californicum* (Table 1). Chemical structures of *V. californicum* steroidal alkaloids may be found in Figure 1 and are identified by a number in bold font. The structures of additional alkaloids described in this review may be found in Figure S1.

Interest in the *Veratrum* genus is attributable to the medicinal and therapeutic properties of steroidal alkaloids produced by the plants. Over 100 alkaloids have been identified, mostly from extraction of the root and rhizome, with several of the alkaloids demonstrating cancer suppression, induction of bradycardia, analgesia, and other effects [1]. Bioactive components from *Veratrum* have been identified within 16 species, but only *V. album*, *V. viride*, and *V. nigrum* have been thoroughly studied [3]. A brief overview of *Veratrum* spp. from which researchers have identified bioactive constituents for potential use as phytotherapeutics is provided. Much of our understanding of *Veratrum* spp. component activity originates from animal or human poisoning associated with accidental ingestion of plant material. Observations of one-eyed sheep birth defects, low heart rate, vomiting, diarrhea, and other side effects attributable to *Veratrum* poisoning have led to investigations identifying active constituents consisting of alkaloid and non-alkaloid compounds. To provide a background of instances of *Veratrum* poisoning that have led to studies of active components and ultimately alkaloid (and non-alkaloid) identification, several case studies are included in this review.

### 1.1. V. album: Subspecies V. lobelianum, V. grandiflorum and V. oxysepalum

*V. album* is a species complex of three subspecies, *V. lobelianum, V. grandiflorum*, and *V. oxysepalum*, sharing the common name white false hellebore [1]. This species is prevalent in northern Eurasia and to a lesser extent may be encountered in localized outcrops in North America, specifically in northern Alaska [1,2]. This plant complex had been used medicinally for centuries for its emetic properties well before the cause of this effect was understood. In 1820 *V. album* became one of the first *Veratrum* species analyzed for the presence of steroidal alkaloids. Eventually it was determined that certain *V. album* alkaloids induced a hypotensive effect by binding to voltage-gated sodium channels, causing the channels to remain open. When a neuronal stimulus occurred, multiple signals were released, because the cell’s ability to repolarize was inhibited. This property led to increased use of the plant to treat hypertension. With rising demand, more plants had to be grown, but the supply was unreliable due to slow growth and germination rates as well as chilling requirements. Phytotherapies incorporating *V. album* alkaloids were also discontinued due to difficulty in achieving the appropriate dosing for patients; the difference between toxic and therapeutic doses was about 30% [1].

#### 1.1.1. *V. lobelianum*

*V. lobelianum* grows in most countries in northern Asia, but is also found less frequently in parts of Europe [4]. In Russia it is called “chemeritsa” and has been used as part of Russian traditional medicine for hundreds of years. A tincture made from the plant has been incorporated as an ingredient in ointments that are rubbed onto a patient’s skin to treat scabies and neuralgic and rheumatic pain. When the roots and rhizomes are boiled and concentrated, they are applied to the scalp as a treatment for lice. Together, baked roots, rhizomes, and cream have been an applicant for eczemas. Aqua veratri, commonly known as Hellebore water, is a dilution of the *V. lobelianum* tincture used to treat psoriasis by rubbing the ointment into the scalp of the patient. *V. lobelianum* has also been consumed to reduce fever, typhus, and pneumonia. The plant is possibly best known as an antiparasitic, including its use to combat hypodermatosis in cattle and to induce emesis in pigs [43].

#### 1.1.2. *V. grandiflorum*

*V. grandiflorum,* also known as white hellebore [3], has a geographical growth region predominantly in Asia [1,2]. Arthritis and gout have been treated using *V. grandiflorum* extract, which contains active components including stilbenoid phenol and resveratrol, which promote anti-inflammatory and anti-osteoarthritis responses [16]. In the 5th century BCE, it was thought that gout was caused by overeating, and thus Hippocrates recommended consuming large amounts of *V. grandiflorum* in order to induce vomiting [8]. In the 1800s, a controversial treatment for gout was a tincture made from a combination of *V. grandiflorum*, tobacco, foxglove, wild cucumber, and a mix of other plants for flavor and aroma [9]. Resveratrol, the component of red wine that has gained considerable attention in recent years, was first isolated in 1940 from *V. grandiflorum*, a fact that is still evident in its name. Resveratrol has had a long history of clinical trials utilizing different diets and dosages of oral supplements to most effectively employ its anti-oxidative, anti-aging, anti-cancer, and cholesterol lowering properties [12,44].

#### 1.1.3. *V. oxysepalum*

The final member of the *V. album* complex is *V. oxysepalum,* which primarily grows in parts of Europe and northeastern Asia [18,19]. *V. oxysepalum* is the most common member of the *V. album* complex to be mistaken for an edible plant; when consumed, it induces poisoning due to the presence and abundance of toxic alkaloids. When *V. oxysepalum* is ingested, a person will suffer from vomiting, a drop in blood pressure and heart rate, and a tingling sensation in their mouth [18]. There have been at least seven situations where *V. oxysepalum* was confused for an edible wild plant, and upon ingestion led to accidental poisoning. The first report was associated with gentian wine, which requires the European *Gentiana lutea*, a plant that bears remarkable similarity to *V. oxysepalum* [18]. In 2004, two men living in Italy consumed a beverage freshly prepared by a friend, thinking it was made from *Gentiana lutea*. Within the hour, they experienced nausea, vomiting, and headaches, and one man had diarrhea. After admittance to the hospital, both patients were treated with activated charcoal and an anti-emetic and discharged within 24 h [45]. A second, similar incident involving confusion of *V. oxysepalum* for *G. lutea* occurred in 2008 [20]. A set of four separate cases occurred in the hills near Ljubljana, Slovenia in the months of April and May 2009 where four adults mistook *V. oxysepalum* for the edible *Allium ursinum* (wild garlic) [21]. In the final reported incident 11 children at a youth camp poisoned themselves by accidentally consuming small amounts of *V. oxysepalum* while attempting to prepare herbal tea [22].

### 1.2. V. maackii

*V. maackii* is native to Asia, commonly encountered in the Henan, Jilin, and Shandong provinces of China. *V. maackii* is used in the Chinese traditional medicine Lilu, which gains its alleged therapeutic benefits from alkaloids, stilbenes, flavonoids, phenols, and glyceride. *V. maackii* has been used for thousands of years to treat symptoms of aphasia caused by jaundice, scabies, apoplexy, and other such ailments. The plant components are teratogens, which is consistent with other *Veratrum* alkaloids, and there are at least nine stilbenes (1. *cis*-mulberroside A, 2. resveratrol-4,3′-*O*-β-D-diglucopyranoside, 3. mulberroside A, 4. gentifolin K, 5. resveratrol-3,5-*O*-β-d-diglucopyranoside, 6. oxyresveratrol-4′-*O*-β-d-glucopyranoside, 7. oxyresveratrol-3-*O*-β-d-glucopyranoside, 8. oxyresveratrol, and 9. resveratrol) that have been identified as bioactive constituents. Stilbenes have anti-oxidant and anti-radical properties that can lead to prevention of cancer and cardiovascular disease as well as anti-inflammatory and neuroprotective effects. The anti-oxidant properties of *V. maackii* stilbenes have been demonstrated in mice through the reduction of ethanol-associated single-stranded DNA breaks [25].

### 1.3. V. nigrum

*V. nigrum*, commonly referred to as black false hellebore, is another plant that grows in Europe and Asia. This particular species of *Veratrum* has been used in both Chinese and Korean medicine. *V. nigrum*, along with a few other species of *Veratrum,* make up the Chinese drug “Yeo-Ro”, which has been used for treatment of headache, jaundice, chronic malaria, dysentery, scabies, and hypertension [28]. In Traditional Korean Medicine, *V. nigrum* is fed to patients to induce vomiting as a treatment for dyspnea caused by a stroke or epilepsy [29]. The alkaloids **2**, **7**, and epi-verazine have been extracted and used as melanogenesis inhibitors in mice, but the mechanism of action remains undetermined [28]. *V. nigrum* is used in China to alleviate aphasia in the same way as *V. maackii,* and also to treat symptoms of hypertension [29,30]. Veratramine has a hypotensive effect by blocking sodium ion channels and acting as an agonist for serotonin on presynaptic 5-HT neurons [30].

### 1.4. V. taliense

*Veratrum taliense* grows in Europe and Asia, and in the Middle Ages was used in both places to treat high blood pressure, excess of phlegm, epilepsy, and stroke. The alkaloids contained within the plant inhibit voltage gated sodium ion channels. Two examples include **3** and rubijervine, which can block Na_V_1.5 channels in cardiovascular muscles and induce cardiotoxicity at elevated levels [32].

The roots and rhizomes of *V. taliense* are a component of the traditional folk medicine “Pimacao” from the Yunnan province of China. The other constituents of Pimacao are *V. grandiflorum*, *V. mengtzeanum*, and *V. stenophyllum*. Pimacao is the main ingredient in “Yunnan Baiyao”, a well-known traditional Chinese medicine for the treatment of pain caused by fractures, hemorrhage, epilepsy, and rheumatism. The mechanism of action has not been thoroughly studied because the formula for this medicine has not been disclosed [13,14,15,46]. Yunnan Baiyao is commonly used for orthopedics, respiratory care, gastroenterology, and gynecology, but some possible adverse drug reactions include abdominal pain, chest tightness, nausea, vomiting, perturbation, and urticaria, among others [46].

### 1.5. V. viride: Subspecies V. viride and V. eschscholtzii

*V. viride* is a species complex of *V. viride* and *V. eschscholtzii*.

#### 1.5.1. *V. viride*

Growing widely across North America, *V. viride,* also known as green false hellebore, was used by some Native American tribes to treat a variety of infirmities and ailments. For a cold, one would chew on the root; for a snake bite, crushed rhizome was applied to the wound; and for venereal disease, a tea made from the plant was consumed. The known ability of *V. viride* to induce vomiting was also used to determine a worthy leader in Native American tribes, based on who could resist the emetic properties the longest. As early as 1835, *V. viride* was consumed to treat rheumatism and as an anti-inflammatory [1]. For those with primary hypertension it has also been shown to reduce high blood pressure by consumption of powdered roots and rhizomes. The alkaloids germidine and germitrine are responsible for the hypotensive properties. Germidine is a diester of the known compound germitrine in combination with acetic acid and *l-α*-metylbutyric acid, whereas germitrine is a triester of the same composition, but with the addition of *d-*methylethylglycolic acid. Germitrine, the more effective constituent, was reported to cause a drop in blood pressure in an anesthetized dog with as little as half a microgram per kilogram administered intravenously [34].

#### 1.5.2. *V. eschscholtzii*

The second member of the *V. viride* subspecies complex is *Veratrum eschscholtzii* [1]. *V. eschscholtzii* is localized in western North America, stretching from California to Alaska. The Native American tribes Bella Coola, Cowlitz, Kwakiutl, Okanagan, Quinault, Salish, Shuswap, and Thompson treated colds and blood, heart, orthopedic, and skin ailments with this plant as well as using it as a poison, analgesic, anti-rheumatic, emetic, and laxative [36]. For example, it was suggested that a poultice of leaves be applied to the body for treatment of pain, or a decoction of the whole plant be ingested for treatment of rheumatism [37]. Isorubijervosine, the glycoside of **3**, was first isolated from this plant along with the already known alkaloids, pseudojervine and **6** [38].

### 1.6. V. californicum

*V. californicum* var. *californicum* is also referred to as California false hellebore. It too grows in the western United States but is often found slightly further south. *V. californicum* was not traditionally used for medicinal purposes, but instead was noticed due to its teratogenic effects. During the 1950s in the high mountain meadows of Idaho, lambs were born with a singular eye in a cyclopean malformation. It was determined that this was caused by the pregnant ewes consuming *V. californicum* before giving birth to offspring. The alkaloids within the plant were determined to be responsible for the interruption of the hedgehog (Hh) signaling pathway retarding the development of the embryo, causing improper tissue formation [1].

Each of the above listed *Veratrum* spp. has been studied because each contains at least one known or suspected alkaloid found within *V. californicum*. There remain many gaps in the research of *V. californicum* as most of the studies were conducted over fifty years ago; thus, the bioactivity source(s) within each additional *Veratrum* spp. were examined in order to provide more information regarding the possible uses of *V. californicum*. The alkaloids found in *V. californicum* have potential to be developed into cancer therapeutics due to their Hh signaling inhibitory properties [40]. In fact, **1** has been used as a molecular template to inspire the development of FDA-approved and clinical trial candidate chemotherapeutics, including glasdegib, saridegib (IPI-926), vismodegib, and sonidegib [47]. Ethanolic extracts of below-ground parts of *V. californicum* were examined using high pressure liquid chromatography (HPLC) and mass spectrometry (MS) analysis, revealing the existence of at least 16 unique components (Table 2). Six of the alkaloids have confirmed identities (green highlight), while five others are proposed (yellow highlight) and another five are completely unknown (red highlight) [41]. The remainder of this review will focus on the medicinal relevance, extraction method, characterization, and bioactivity of each of the eleven known and proposed alkaloids.

## 2. Alkaloids Identified in *Veratrum* *californicum*

For each *V. californicum* alkaloid that is known or proposed, the following section details how these components were characterized based on three criteria: (1) method of extraction and isolation, (2) process of identification, and (3) reported bioactivity. The eleven best characterized alkaloids in *V. californicum* (see Figure 1; green and yellow entries from Table 2) were first identified from a variety of different plant species, so a description of the original discovery has been detailed. In instances where two or more procedures for extraction from the same species of plant provided the same alkaloid, two methods have been described: the original and the most up to date refined protocol. They are presented in the order of alkaloids with a confirmed presence followed by the suspected alkaloids. An overview of the methods by which each *V. californicum* alkaloid was extracted and its properties and potential medicinal uses are summarized in Table 3 and Table 4.

### 2.1. Cyclopamine *(**1**)*

A modern method of **1** extraction from *V. californicum* in 2013 included the use of a Soxhlet apparatus [1]. Biomass from root and rhizome was ground to a fine powder and wetted with 2 mL of 98.3:1.7 Ethanol:Ammonioum Hydroxide (*v*/*v*) solution and then packed into a cellulose extraction thimble. The contents of the thimble were extracted in a Soxhlet apparatus with two 50 mL portions of 98.3:1.7 EtOH:NH_4_OH over consecutive 12 h segments. The extract was subjected to rotary evaporation and stored at −20 °C [1]. Beginning with crude alkaloid mixtures, **1** can be separated via HPLC using a C_18_ column [48,49].

Compound **1** can be quantified through LC chromatograms by first generating a calibration curve from the commercially available standard, then comparing areas under the curve to experimental values. Once isolated, **1** and its derivatives can be characterized by MS [48,49]. A full list of 700 MHz proton and carbon-13 chemical shift assignments for **1** characterization is available in literature [50].

Compound **1** and its derivatives have been shown to inhibit the Hh signaling pathway. Active during embryonic development, the Hh pathway plays an important role in limb patterning and specification of cells in the nervous system. Typically dormant in cells after embryonic development, aberrant activation of Hh can result in tumorigenesis [51,52]. Compound **1** was the first identified molecule to inhibit the Hh signaling pathway [53]. The activation of the Hh pathway requires signal transduction by the transmembrane protein smoothened (Smo) [54]. Compound **1** inhibits the Hh signaling pathway by blocking smoothened after it binds to an internal helical fold of the transmembrane portion of the receptor protein [55]. In most adult tissues, the Hh signaling pathway is inactivated [56]. Aberrant Hh activation can lead to oncogenesis, but **1** and its derivatives are promising therapeutic agents that can be used for treatment. Two such derivatives are KAAD-cyclopamine and saridegib [57,58]. Both have higher potency and stability compared to **1**. In addition to targeting Smo and blocking Hh signaling, **1** can also interfere with myriad other harmfully mutated biological processes: it has been shown to inhibit growth of breast cancer and erythroleukemia cells through mechanisms outside of Smo binding [59,60], and has been shown to induce apoptosis in human prostate cancer cells [61]. Tumor necrosis factor (TNF)-related apoptosis-inducing ligand (TRAIL) is an extremely useful cancer treatment that kills only cancerous cells without disturbing normal cells. Cancer cells located in the stomach are resistant to TRAIL due to an absence of death receptor 5. Compound **1** causes increased expression of death receptor 5 in TRAIL-resistant gastric cancer cells, making them more susceptible to TRAIL [62]. Outside of cancer cell suppression, **1** blocks transcription of human respiratory syncytial virus both in vitro and in vivo and also holds some promise in future treatment of psoriasis by promoting healing of psoriatic skin lesions [63,64].

**Table 3:** Methods of extraction, separation, and identification of *Veratrum* alkaloids.

Alkaloid	Plant(s)	Sample Preparation *	Separation Technique	Identification	References
Cyclopamine (**1**)	*V. californicum*	Soxhlet extraction with ethanol/ammonium hydroxide	HPLC	LC-MS, ^1^H and ^13^C NMR	[1,50]
		Ethanol soak			[48,49]
Veratramine (**2**)	*V. viride*	Ethanol and chloroform extraction	Flash chromatography with silica gel	^1^H and ^13^C NMR, HPLC-MS, crystallization, melting point, and HPLC-CAD.	[65]
	*V. viride*	Benzene, ammonia, acetic acid, and NaOH extractions	High-speed counter-current chromatography	HPLC, MS and NMR	[66]
	*V. oxysepalum*	Diethyl ether and dichloromethane extractions	N/A	HPLC-MS	[67]
	*V. nigrum* L.	Ethanol and then chloroform extraction	Column chromatography	Crystallization	[68]
	*V. grandiflorum*	Crystallization and filtration using 2 N-calcium acetate and acetic acid (Unk plant part)	N/A	Crystallization, melting point, and NMR	[69]
	*V. californicum*	Dried and ground, ethanol then chloroform extraction	HPLC	HPLC, CAD and MS	[70]
Isorubijervine (**3**)	*V. eschscholtzii Gray*	Chloroform extraction (Unknown plant part)	Craig countercurrent distribution	N/A	[71]
	*V. taliense*	Methanol extraction	Silica gel column chromatography and MPLC	NMR and ESI-MS.	[32]
	*V. viride Aiton*	Ethanol and chloroform extraction (Unk plant part)	Flash chromatography with silica gel	IR, LC-MS, ^1^H and ^13^C NMR	[65]
Muldamine (**4**)	*V. californicum*	Benzene and 5% NH_4_OH soak (Unk plant part)	Column chromatography with silica gel/benzene/methanol slurry	HPLC-ELSD, MS *m*/*z* = 458.37	[72]
Cycloposine (**5**)	*V. californicum*	Dried and ground, ethanol then chloroform extraction	HPLC-ELSD	HPLC-MS spectra confirmation	[70,72,73]
Veratrosine (**6**)	*V. patulum*	Ethanol soak, chloroform extraction, alkaloid residue	Silica gel, recrystallization in acetone	HPLC-MS *m*/*z* and elution times	[70,72,73]
	*V. californicum*	Dried and ground, ethanol then chloroform extraction	HPLC-ELSD		
Verazine (**7**)	*Zygadenus sibiricus*	N/A (From aerial plant)	N/A	N/A	[74]
	*Solanaceae Solanum hypomalacophyllum*	Dried, ground and refluxed with CHCl_3_	Vacuum liquid chromatography and column chromatography	N/A	[75]
	*Asteraceae Eclipta alba*	EtOAc and MeOH extraction from leaves	Silica gel columns and preparative TLC	MeOH crystallization and DEPT, HETCOR, HMQC, and HMBC NMR	[76]
	*V. nigrum*	Ethanol extraction followed by two CHCl_3_ extractions (Unknown plant part)	Alkali-treated silica gel and TLC	Specific TLC fractions were recrystallized	[77]
	*V. nigrum*	Three methanol extractions	Silica gel column chromatography twice and HPLC	Recrystallization, ESI-MS, IR, ^1^H and ^13^C NMR	[28]
Etioline (**8**)	*Solanum spirale*	Dried and ground, Soxhlet extraction with ethanol, chloroform/methanol extraction	Silica gel	^13^C NMR and atom probe chromatography	[78]
	*Lilium candidum* L.	Ethanol extraction from bulbs and then CHCl_3_ extraction	TLC with chloroform and methanol	TLC with chloroform and methanol	[79]
Tetrahydrojervine (**9**)	N/A	Synthesized only			
Dihydrojervine (**10**)	N/A	Synthesized only			
22-Keto-26-amino cholesterol (**11**)	N/A	Cholesterol metabolite, proposed			[80,81]

### 2.2. Veratramine *(**2**)*

Compound **2** has been extracted from many different *Veratrum* spp., including *V. viride* [65,66], *V. oxysepalum* [67], *V. nigrum* L. [68] and *V. grandiflorum* [69]. One method of extraction from *V. viride* involved percolation with 95% ethanol, followed by evaporation, resuspension in 5% tartaric acid, and filtration. Additional percolation of the filtrate was performed with chloroform, where the extract was subjected to pH adjustment and solvent evaporation. Flash chromatography with silica gel was used to separate **2** from other alkaloids [65]. Another method of extraction from *V*. *viride* involved grinding the roots and rhizomes to a powder, and then performing extractions using benzene and dilute ammonia. Additional extractions were performed with acetic acid, NaOH, and again with benzene until a crude alkaloid mixture was produced. In order to purify **2** from the mixture, ammonium sulfate was added and the resulting mother liquor was diluted with ammonia until the alkaloid could be removed with 50% ethanol [66]. An alkaline solution of powdered *V. oxysepalum* biomass was refluxed with ethanol and filtered. Additional extractions of the filtrate with diethyl ether and dichloromethane were performed, resulting in a crude extract of alkaloids. The isolation of **2** from *V*. *oxysepalum* was carried out via a two-step use of high-speed counter-current chromatography (HSCCC) [67]. In order to remove the alkaloids from *V*. *nigrum* L., extractions were first performed with 95% ethanol. After concentration, the pH was lowered to 3 using hydrochloric acid, and then the solution was filtered. The pH of the filtrate was raised to 10 using ammonium hydroxide, and additional extractions of crude alkaloids were performed with chloroform. Separation of alkaloids was achieved via column chromatography [68]. The resinous material of *V*. *grandiflorum* passed through a series of crystallizations and filtrations over a period of several days. The mother liquor from the crystallization of jervine hydrochloride was dissolved in 0.5 N-acetic acid, and 2 N-sodium sulfate was added, eventually leading to formation of veratramine sulfate and then **2** [69].

After extraction from the roots and rhizomes of *V. viride*, **2** was characterized in different solvents using ^1^H and ^13^C NMR [82]. After **2** was separated from a crude extract of *V. oxysepalum*, the data from HPLC, MS, and NMR were analyzed to confirm its identity [67]. A C_18_ column was used for HPLC-MS isolation of **2** from V. *nigrum* L. This was followed by ESI-MS analysis in a *m*/*z* range of 200–700 amu [83]. The alkaloid quantity from *V. californicum* was determined using HPLC with a charged aerosol detector (CAD) and MS via calibration curves that were determined in triplicate in accordance with a purchased standard (**2**). Identification of the alkaloid was performed with HPLC-MS [70]. Melting point has been used for characterization, as colorless needles of **2** melt at 209.5 to 210.5 °C. The specific rotation values were found to correspond closely to calculated values. The mother liquor of a crude jervine hydrochloride from *V. viride* had excess ammonia added until separation of **2** was achieved. The product was recrystallized and jervine contaminants were removed to achieve the same melting point as above. In order to confirm that **2** had indeed been produced, the product was acetylated and compared to triacetylveratramine, and was found to possess the same properties and melting point. Specific rotation values were also calculated and verified with experimental results [66].

Compound **2** has been tested for inhibition of prostate cancer by interfering with the Hh signaling pathway, and in conjunction with other alkaloids, has been used for treating high blood pressure, apoplexy, and other ailments [82,83]. Bioactivity of **2** was measured in various ways. The ability of **2** to inhibit the growth of cancerous cells on a malignant PC-3 cell line was tested, and a wound-healing assay was also performed. Out of nine alkaloids tested in the wound-healing assay, **2** and its derivatives exhibited the highest degree of anti-migratory activity, and at a concentration of 50 µM, **2** displayed the highest degree of prevention of proliferation of cancerous cells [82]. In another study, **2** was introduced to two different groups of rats by gavage at doses of 0.25 or 2.50 µmol/kg for seven days to test its neurotoxicity both in vivo and in vitro using rat liver microsomes. DNA was damaged in the cerebellum and the cerebral cortex at both high and low doses of **2** as detected using the alkaline comet assay [83].

### 2.3. Isorubijervine *(**3**)*

Compound **3** has been found in many different species of *Veratrum*. Isolation of **3** from *V. eschscholtzii* Gray was achieved by crude chloroform extraction. A Craig countercurrent distribution was performed on this extract to separate the alkaloids and identify **3** [71]. In a separate study, the roots and rhizomes of *V. taliense* were dried and ground before being extracted with methanol. After solvent acidification, the extract was filtered, and then base was added before silica gel column chromatography (CC) was utilized for component separation. The second eluted fraction underwent medium pressure liquid chromatography (MPLC) to yield **3** [32]. This alkaloid was also obtained from *V. viride* Aiton through a series of ethanol and chloroform extractions. The identification of specific alkaloids was made possible after flash chromatography was performed using silica gel [65].

The amount of **3** contained in the extract from *V. californicum* was quantified by creating a calibration curve for HPLC using commercially available **3**. In order to determine measurement of **3**, the HPLC was attached to a CAD and an MSQ plus MS [70]. Compound **3** was identified from the extract of *V. taliense* using NMR and ESI-MS [32]. The melting point of the colorless needles of isorubijervine was found to be 241–242 °C. IR, LC-MS, and ^1^H and ^13^C NMR were used to characterize this compound [65].

The traditional medicinal benefits of this alkaloid include the treatment of pain and hypertension, but the mechanism remains unknown [30,32]. Compound **3** has been used to treat high blood pressure and pain, but has also been found to cause toxicity to the heart. The LD_50_ of **3** was found to be 1.14 mg/kg in mice. Administration of **3** to rats and macaques resulted in bradycardia and hypotension that lasted for several minutes; the response observed in macaques was dose-dependent. Na_v_1.5 sodium channels expressed in HEK293t cells, which are critical to cardiovascular function, were treated with **3**. The IC_50_ value was found to be 6.962 ± 0.422 µM, and a 5 µM dose led to a 41% decrease in current. This decrease in flow in the sodium channel may explain the symptoms of bradycardia in the animals [32].

### 2.4. Muldamine *(**4**)*

Compound **4** was extracted by soaking *V. californicum* in a benzene/5% NH_4_OH (3:1 *v*/*v*) solution for 24–48 h. This solution was then dried in vacuo to produce a mixture of alkaloids that were recrystallized first in an acetone/water solution and then in a methanol/water solution. The recrystallized alkaloid mixture (300 mg) was dissolved in benzene/methanol (10:1, 3 mL) and loaded onto a chromatography column packed with a slurry of silica gel and benzene/methanol (60:1). Elution was performed using a benzene/methanol solution (60:1, 20:1). Isolation of **4** was performed using HPLC-ELSD in combination with manual fraction collection of individual peaks [72].

Compound **4** has been characterized by a *m*/*z* of 458.37 [72]. The identity of **4** was verified using a commercially available standard.

Initially given the name alkaloid Q, **4** is produced by *V.* *californicum* and is known to be a Hh signaling pathway antagonist as well as a non-depolarizing action potential blocker in the giant axon of squid and crayfish [1,73,80]. Compound **4** is not teratogenic in sheep, but did demonstrate marginal activity as a teratogen in hamsters [81]. The bioactivity of **4** was tested in combination with **2** and **3** by measuring Gli protein activity in Shh-Light II cells [73]. The Gli family members are transcription factors that can be monitored in order to determine whether the Hh pathway is activated or not [84,85]. The results demonstrated that the combination of alkaloids did have an inhibitory effect on Hh signaling [73].

### 2.5. Cycloposine *(**5**)*

In a 2019 study by Turner et al., **5** was obtained by first cutting the rhizome/root of *V. californicum* into 1–2 cm cubes and lyophilizing it to dryness over 24–48 h. The dried biomass was then submerged in liquid nitrogen and ground to a fine powder using a mortar and pestle. Powdered biomass (2.0 g) was added to 95% ethanol (100 mL) and sonicated for 30 min, then mixed on a stir plate for 24 hrs. Plant material was removed via vacuum filtration and discarded. Ethanol was separated from the resulting solution by rotary evaporation, yielding a brown oil. The crude alkaloid product was dissolved in 95% ethanol (10 mL), warmed to 40 °C, and sonicated to ensure all alkaloid product was dissolved. An aqueous ammonia solution (35% *v*/*v*) was added to obtain an alkaline solution (pH ≥ 10). This solution was eluted through a supported liquid extraction column with chloroform (3 × 10 mL) using a vacuum manifold (2 mbar). The fractions were combined, filtered and dried. The dried alkaloids were then dissolved in 100% ethanol [70]. HPLC-ELSD was used to separate individual alkaloids from the mixture [72].

Compound **5** was identified by HPLC-MS by comparison to a commercially available standard [70,73].

Compound **5** is a teratogenic alkaloid produced exclusively in *V. californicum* [1]. This alkaloid is relevant to medicine through its use in the development of modern cancer treatments [1]. Shh-Light II cell models demonstrated that **5** did not contribute to Hh signaling inhibition in this method of bioactivity analysis; however, deglycosylation from hydrolysis during digestion may contribute to teratogenicity [73].

### 2.6. Veratrosine *(**6**)*

In 1998, researchers conducted an extraction of *V. patulum* by cutting the roots into small pieces and soaking them in ethanol (4 × 7 L). The four ethanol solutions were concentrated in vacuo to form a residue that was dissolved in 5% aqueous tartaric acid solution (2.5 L); insoluble materials were removed by filtration. The solution was defatted with ether (4 × 3 L) and made alkaline with 20% Na_2_CO_3_ to pH 10. Extraction was performed with CHCl_3_ (4 × 500 mL), where all chloroform extractions were combined and dried to form an alkaloid residue [86]. The alkaloid components in the *V. patulum* extract were separated by column chromatography using alkali-treated silica gel (400 g) and a mobile phase of MeOH-CHCl_3_ (2:98, 6:94, 10:90, and 15:85). Thin layer chromatography (TLC) was used to monitor eluates with a total of 26 fractions collected. Fraction 23 was recrystallized with acetone to yield **6** (mp 242–244 °C) [86].

In 2013, **6** was identified from a *V. californicum* ethanol extract using HPLC-ELSD [72]. Most recently, the identification of **6** has been accomplished using HPLC-MS and verified by comparing the elution time of a commercially available standard [70,73].

When ingested, **6** is an antagonist of epinephrine and norepinephrine [87]. In Shh-Light II cell models it was shown that **6** did not contribute to Hh signaling inhibition; however, hydrolysis of the glycosidic linkage during digestion may contribute to teratogenic effects [73].

### 2.7. Verazine *(**7**)*

Compound **7** has been extracted from different plant families including *Lilaceae* in both species *Veratrum* (*nigrum*) and *Zygadenus* (*sibiricus*), *Solanaceae Solanum* (*hypomalacophyllum*), and *Eclipta* (*alba*) [28,75,76,77]. The roots of *Solanum hypomalacophyllum* were dried and ground to a powder before 10% NH_4_OH was added. The basic mixture was refluxed with CHCl_3_ and dried. Fractions were collected after using vacuum liquid chromatography and various forms of column chromatography with CHCl_3_-MeOH and H_2_O-MeOH gradients, resulting in both epimers of **7** (20S and 20R) [75]. Dried leaves from *Asteraceae Eclipta alba* were extracted using both EtOAc and MeOH. A silica gel column was used with Me_2_CO in CHCl_3_ to elute fractions, which were purified using a second silica gel column and preparative TLC. Compound **7** was also crystallized from MeOH [76]. Extraction from the roots and rhizomes of *V. nigrum* L. var. *ussuriense* was done using ethanol after the plant matter had been dried and portioned into small pieces. The extract was concentrated in vacuo before the addition of a 5% aqueous tartaric acid solution. After the sample was filtered, ether was added, and then another extraction was performed using CHCl_3_. A 20% aqueous Na_2_CO_3_ solution was added to the aqueous portion until a pH of 6 was achieved. Another CHCl_3_ extraction was performed, and the product was dried before separation by chromatography using alkali-treated silica gel with different concentrations of mobile phase MeOH-CHCl_3_. TLC separated the fractions, and fractions 5–7 were combined and recrystallized from acetone yielding **7** [77]. Another extraction from the crushed roots of *V. nigrum* was performed using methanol, repeated three times. The extract was diluted with water before being separated into different layers using n-hexane, chloroform, and butanol. The butanol layer underwent silica gel column chromatography twice, first using acetonitrile-methanol, and then using n-hexane-acetone-methanol. Finally, three different compounds were isolated using HPLC [28].

Compound **7** is usually in the form of colorless needles, and the melting point is between 175 and 177 °C. High resolution ESI-MS was used in order to identify the molecular weight as 397.65 amu and the molecular formula as C_27_H_43_NO [28]. Compound **7** is typically characterized via NMR and sometimes IR. The spectra are well established, so the presence of **7** can be confirmed by spectral comparison [88]. Important IR, ^13^C and ^1^H NMR chemical shifts are those of the alcohol, imine, specific methyl groups, and singular hydrogen and carbon atoms, which have all been described [28]. Assignments of chemical shifts have also been obtained using a variety of NMR experiments including DEPT, HETCOR, HMQC, and HMBC [76]. Deuterochloroform was used as the solvent for the 1D ^1^H and ^13^C and 2D double quantum filter COSY and NOESY NMR spectra of **7** [89].

The medicinal properties of **7** have been examined for use as an antifungal agent or a potential melanogenesis inhibitor, but the mechanism of action is still being explored [28,76,81,90]. Most **7** bioactivity appears to be antifungal. In one study, steroidal alkaloids were used as inhibitors of *Candida albicans* and *Trichophyton* spp.; **7** was a successful inhibitor with a minimum inhibitory concentration of 6.2 µg/mL for *C. albicans* and 3.1 µg/mL for *T. rubrum* [90]. Yeast assays using the Sc7 yeast strain were inhibited by **7**, but when examining cytotoxicity, the results were not as favorable; IC_50_ values were greater than 10 µg/mL. Due to this cytotoxicity, antifungal studies were aborted [76]. Compound **7** was also studied for potential use as a melanogenesis inhibitor. The IC_50_ was less than 1 µg/mL for inhibiting melanin biosynthesis in B16 FI mouse melanoma cells [28].

### 2.8. Etioline *(**8**)*

Compound **8** was extracted from the root of *Solanum spirale*. The roots were heated, dried, and ground to a powder prior to undergoing a Soxhlet extraction with ethanol. The ethanol was evaporated under vacuum and partitioned evenly between 10% C_6_H_6_-Et_2_O and HOAc. Ammonia was added, and a second extraction was performed with CHCl_3_-EtOH. The solvents were again evaporated, and silica gel was used to obtain an elution of **8** with CHCl_3_-MeOH [78]. Compound **8** has also been extracted from *Lilium candidum* L. bulbs using ethanol. This extract was combined with HCl for three days before the pH was raised using ammonia, and the resulting aqueous portions were extracted with CHCl_3_ [79].

To characterize the *Solanum spirale* extracted alkaloids, analysis by NMR spectroscopy was pursued. The ^13^C NMR spectrum of the alkaloid suspected to be **8** was compared to solafloridine, 20-isosolafloridine and 20,25-bisisoetioline, and supported by atom probe chromatography measurements [78]. The *Lilium candidum* L. extract was examined via TLC with CHCl_3_:MeOH, resulting in the identification of jatropham and 22,26-epiminocholestane-type steroidal alkaloid, which is consistent with **8** [79].

Compound **8** has been tested for the treatment of Hepatitis B, and has proven to be effective in specific contexts [91]. PLC/PRF/5 cells were prepared from human hepatoma, which were constantly excreting hepatitis B surface antigen, while KB cells were of the HeLa cell line and believed to possess human papillomavirus-18 (HPV-18) [91,92]. Compound **8** was applied to these human PLC/PRF/5 and KB cells in vitro, and it was found that it only significantly inhibited the human PLC/PRF/5 cells, showing inhibition of Hepatitis B virus, but not of HPV-18 [91].

### 2.9. Tetrahydrojervine *(**9**)*

Compound **9** results from the reduction of the C5-C6 and C12-C13 double bonds in jervine and has been hypothesized to occur in *V. californicum* due to the presence of jervine [40,93]. In past experiments, **9** was artificially synthesized through the reduction of jervine using PtO_2_ in acetic acid [94].

A physical property that assists identification of **9** is its melting point of 221 °C (decomposition) [94]. Characterization of **9** has involved specific rotation measurements and use of the Zerewitinoff determination to identify active hydrogen atoms for the purpose of aiding structure determination [95,96]. Possible methods of characterizing this alkaloid such as NMR, HPLC, and GC-MS have not been reported in the literature.

Compound **9** has not been used as a medicine, but has been used as a tool to examine the effects of *Veratrum* alkaloids on embryonic development [40]. Studies involving **9** investigated the relative teratogenic potency of jervines and how saturation of the C5-C6 bond led to less severe Hh pathway inhibition [94]. Compound **9** was tested on explants of the intermediate neural plate region of Stage 9–10 chick embryos. Explants were treated with **9** in 48 nM and 240 nM concentrations and tested for induction markers of floor plate (HNF-3β) and motor neuron (Isl1/2) differentiation with the goal of inhibiting their growth. Compound **9** produced only 43% inhibition of HNF-3β at 240 nM [97]. In another study, it was determined that **9** was significantly less teratogenic than jervine, **10**, and **1**. Pregnant Syrian hamsters that were dosed with jervine produced fetuses where 92% possessed malformations, whereas only 14% of fetuses had malformations when exposed to **9** during gestation [94].

### 2.10. Dihydrojervine *(**10**)*

Compound **10** is synthetically obtained by reducing the C12-C13 double bond of jervine using PtO_2_ or LiAlH_4_. The latter yielded a mixture of oils and crystalline products which included **10** as the major one [98].

The successful formation of **10** is confirmed by ^13^C NMR through loss of the double bond signals at 137.2 ppm and 146.4 ppm and a shift of the methyl carbon C21 of jervine found at 12.4 ppm to 10.5 ppm in the spectrum [99].

Compound **10** was tested on the human metastatic prostate cancer PC-3 cell line with wound-healing assays to monitor migration and with 3-(4,5-dimethylthiazol-2-yl)-2,5-diphenyl tetrazolium bromide (MTT) to test proliferation. At a 50 µM concentration, **10** did inhibit cancer growth, but it was ineffective in the wound-healing assay [82].

### 2.11. 22-Keto-26-aminocholesterol *(**11**)*

Compound **11** has not yet been definitively identified from *V. californicum*, but is suspected based on mass spectrum data and a compound library screen [80,81]. Compound **11** is not typically a stable compound and thus will undergo ring-closing to yield **7**; it has not been isolated previously. 22-Hydroxy-26-aminocholesterol can be oxidized by 22-hydroxy-26-aminocholesterol 22-oxidase to form **11** [80,81]. It is a potential intermediate in the biosynthesis of **7** and **1** [81].

A summary of bioactive *V. californicum* alkaloids can be found in Table 4.

**Table 4:** Summary of bioactivity in *Veratrum californicum* alkaloids.

Alkaloid	Method of Testing	Efficacy	Reference
Cyclopamine (**1**)	Inhibition of growth of estrogen receptor positive cell line MCF7	Significant effects at both 10 and 20 µM	[59]
	Inhibition of proliferation of HEL and TF1a cells	Strong effect at 40 µM	[60]
	Induced apoptosis in human erythroleukemia cells	40 µM	[60]
	Inhibition of growth of LNCaP C4-2B cells	Significant inhibition at 100 nmol/L and IC_50_ of 11 µmol/L.	[61]
	Decreased cell viability	<75% viability at 20 µM with 50 ng/mL TRAIL in TRAIL-resistant AGS cells	[62]
	Inhibition of hRSV infection in vitro	IC_50_ of 36 nM	[100]
	Reduction of lung hRSV titers	Reduction by 1.5 logs at 100 mg/kg	[100]
Veratramine (**2**)	Inhibition of progression of human prostate metastatic cancer cell line PC-3	<40% proliferation at 50 µM dose	[82]
	Inhibition of progression of human prostate metastatic cancer cell line PC-3	<20% migration at 50 µM dose	[82]
	Number of DNA-strand breaks in the cerebellum and cerebral cortex of mice	In both cerebellum and cerebral cortex: >0.5 µm tail moment with 0.25 µmol/kg dose, >1.0 µm tail moment with 2.5 µmol/kg	[83]
Isorubijervine (**3**)	Inhibition of rNaV1.3	IC_50_ of 12.14 ± 0.77 µM	[32]
	Inhibition of rNaV1.4	IC_50_ of 9.82 ± 0.84 µM	[32]
	Inhibition of rNaV1.5	IC_50_ of 6.962 ± 0.422 µM	[32]
	Inhibition of rNaV1.5	5 µM dose led to 41% decrease in current	[32]
	Lethal dose in mice	LD_50_ of 1.14 mg/kg	[32]
Muldamine (**4**)	Blocks action potential in squid and crayfish giant axons	Little or no depolarization at 1 × 10^−4^ M	[101]
Verazine (**7**)	Antifungal	Minimum inhibitory concentration of 6.2 µg/mL for *C. albicans*	[90]
	Antifungal	Minimum inhibitory concentration of 3.1 µg/mL for *T. rubrum*	[90]
	Inhibition of melanogenesis in B16 FI mouse melanoma cells	IC_50_ < 1 µg/mL	[28]
Etioline (**8**)	Inhibition of hepatitis B in PLC/PRF/5 cells	EC_50_ of 2.67 µg/mL	[91]
Dihydrojervine (**10**)	Inhibition of progression of human prostate metastatic cancer cell line PC-3	<40% proliferation at 50 µM dose	[82]

## 3. Conclusions

*V. californicum* contains a multitude of bioactive alkaloids with potential to be developed into therapeutic drugs. Compound **1** is an alkaloid from *V. californicum* that has inspired novel Hh pathway inhibiting cancer treatments. There remain many alkaloids in *V. californicum* that have yet to be fully characterized. Advances in separation and identification methods, materials, and instrumentation sensitivity have permitted detection of alkaloids beyond those that have been characterized prior. An assessment of the strategies for extraction, isolation, and characterization of known alkaloids has been presented in an effort to extend existing knowledge of these compounds to alkaloids that have been detected, but not yet characterized (Figure 1, Table 2). Utilization of the methods summarized in Table 3 may permit the identification of additional alkaloids beyond those that are known, so that the identity of all 16 detected components can be accomplished and sufficient quantities of purified alkaloids can be assessed for mechanisms of activity. *Veratrum* plants are a well-known source of medicinal components and the discovery of new compounds in *Veratrum californicum* with favorable therapeutic properties serves as a compelling pursuit.

## Figures and Tables

**Table 1:** Flower and geographic region for *Veratrum* spp. addressed in this review.

Species	Flower	Region	Alkaloid(s)	Other Bioactive Components	Reference
*V. album* var. *lobelianum*	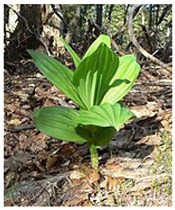	Northern Asia, parts of Europe	Verabenzoamine, veratroilzigadenine, 15-O-(2-Methylbutyroyl)germine, veralosinine, veranigrine, **7**, jervine, pseudojervine, rubijervine, veralosine, veralosidine	Et linoleate, β-Sitosterol, resveratrol, oxyresveratrol	[4,5,6,7]
*V. album* var. *grandiflorum*	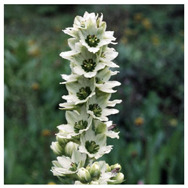	Asia	**2**	Resveratrol	[2,3,8,9,10,11,12,13,14,15,16,17]
*V. album* var. *oxysepalum*	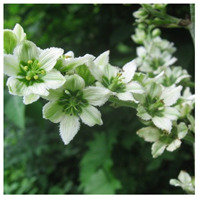	Parts of Europe and northeastern Asia	**2**, veratridine, cevadine		[18,19,20,21,22,23,24]
*V. maackii*	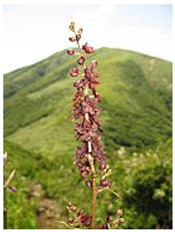	Asia	Verussurine, verabenzoamine, **7**, isoverazine, verazinine, 23-methoxycyclopamine 3-O-β-D-glucopyranoside, isoecliptalbine	Stilbenes, flavonoids, phenols, glyceride	[25,26,27]
*V. nigrum*	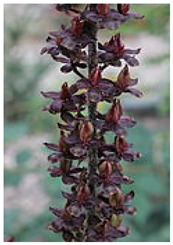	Europe and Asia	**7**, epiverazine, **2**		[28,29,30,31]
*V. taliense*	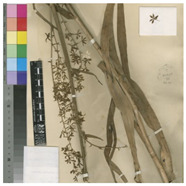	Europe and Asia	Alkaloids including isorubijervine and rubijervine		[13,14,15,32,33]
*V. viride* var. *viride*	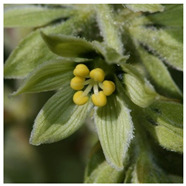	North America	**2**		[1,34,35]
*V. viride* var. *eschscholtzii*	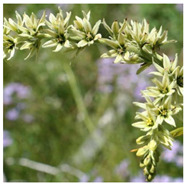	North America	Isorubijervosine, pseudojervine, **6**		[1,36,37,38,39]
*V. californicum* var. *californicum*	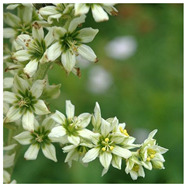	North America	**1**, **2**, **3**, **4**, **5**, **6**		[1,40,41,42]

**Table 2:** Qualitative assessment of *Veratrum californicum* alkaloids.

Identity *	[M+H]^+^ (*m*/*z*)	Predicted Molecular Formula
Cyclopamine (**1**)	412.326	C_27_H_41_NO_2_
Veratramine (**2**)	410.312	C_27_H_39_NO_2_
Isorubijervine (**3**)	414.343	C_27_H_43_NO_2_
Muldamine (**4**)	458.370	C_29_H_47_NO_3_
Cycloposine (**5**)	574.381	C_33_H_51_NO_7_
Veratrosine (**6**)	572.365	C_33_H_49_NO_7_
Verazine (**7**)	398.347	C_27_H_43_NO
Etioline (**8**)	414.342	C_27_H_43_NO_2_
Tetrahydrojervine (**9**)	430.337	C_27_H_43_NO_3_
Dihydrojervine (**10**)	428.320	C_27_H_41_NO_3_
22-keto-26-aminocholesterol (**11**)	416.357	C_27_H_45_NO_2_
N/A	576.396	C_33_H_53_NO_7_
N/A	574.381	C_33_H_51_NO_7_
N/A	576.397	C_33_H_53_NO_7_
N/A	410.311	C_27_H_39_NO_2_
N/A	412.326	C_27_H_41_NO_2_

* Color coding of sections in Table 2 correspond to alkaloids that have been characterized (green), those that have been proposed, but not confirmed (yellow), and alkaloids that have been detected, but are yet to be identified (red).

## Data Availability

Not applicable.

## Supplementary Materials

The following are available online, Figure S1: Structures of *Veratrum* spp. steroidal alkaloids, excluding those found in *V. californicum.*

## Abbreviations

CAD	Charged Aerosol Dectector
CC	Column Chromatography
COSY	Correlation Spectroscopy
DEPT	Distortions Enhancement by Polarization Transfer
ELSD	Evaporative Light Scattering Detector
GC	Gas Chromatography
HETCOR	Heteronuclear Correlation
Hh	Hedgehog
HMBC	Heteronuclear Multiple Bond Coherence
HMQC	Heteronuclear Multiple Quantum Coherence
HPLC	High Performance Liquid Chromatography
HPV-18	Human Papillomavirus Virus-18
hRSV	Human Respiratory Syncytial Virus
HSCCC	High-Speed Counter-Current Chromatography
IC_50_	Half Maximal Inhibitory Concentration
IR	Infrared Spectroscopy
ITS	Internal Transcribed Spacers
KAAD	3-keto N-(aminoethyl-aminocaproyl-dihydrocinnamoyl)
LC	Liquid Chromatography
LDL.	Low-Density Lipoprotein
MHz	MegaHertZ
mp	Melting Point
MPLC	Medium Pressure Liquid Chromatography
MS	Mass Spectrometry
MSQ	Mass Single Quadrupole
MTT	3-(4,5-dimethylthiazol-2-yl)-2,5-diphenyl tetrazolium bromide
NMR	Nuclear Magnetic Resonance Spectroscopy
NOESY	Nuclear Overhauser Effect Spectroscopy
TLC	Thin Layer Chromatography
