# Supplementary material for: Review: Veratrum californicum Alkaloids
Source: Molecules. 2021 Sep 30;26(19):5934. doi: 10.3390/molecules26195934 (PMC8513088; doi:10.3390/molecules26195934)
Supplement: Supplementary file 1 [file molecules-26-05934-s001.zip › molecules-1389857-supplementary.pdf]

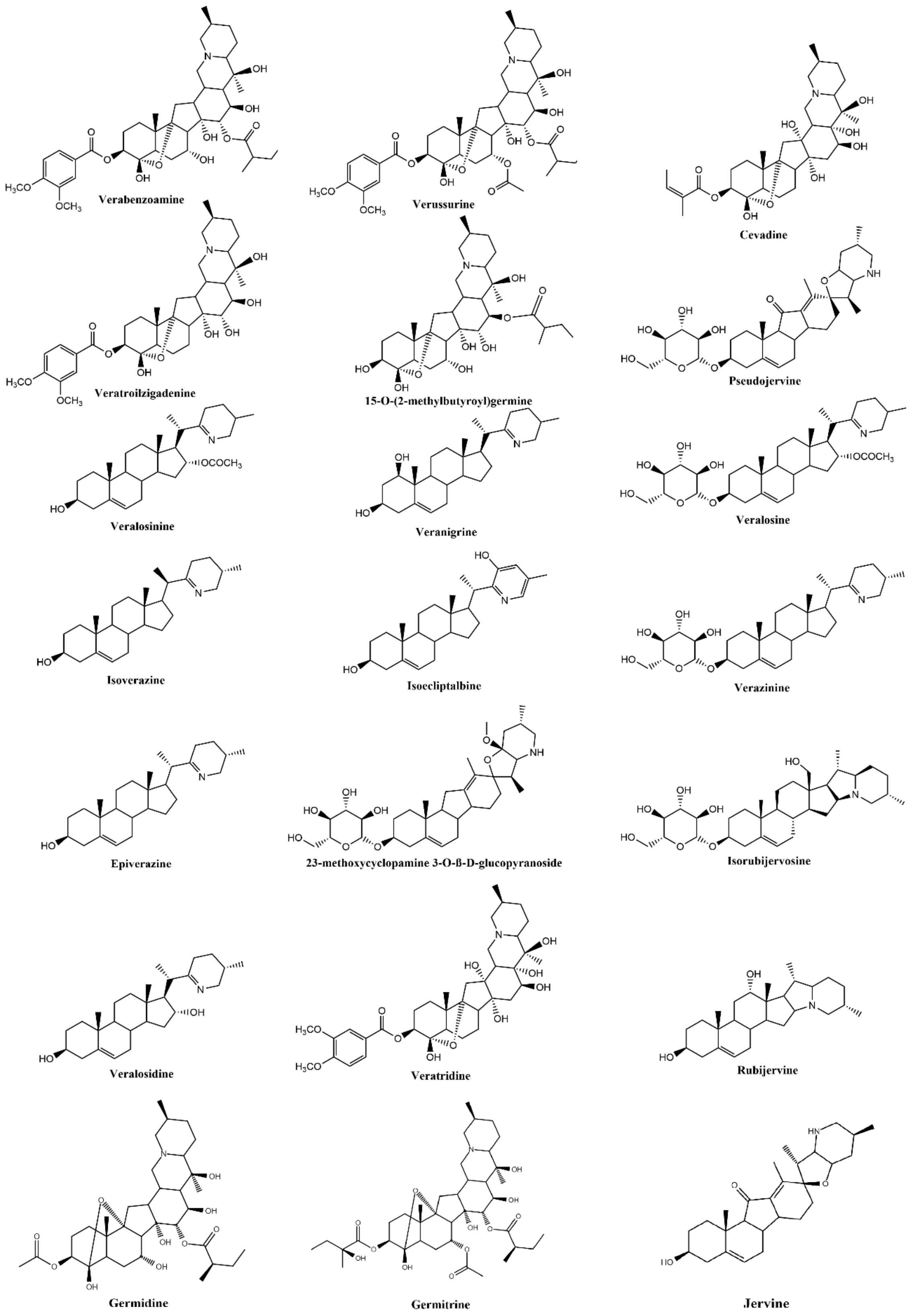

Figure S1. Structures of *Veratrum* spp. steroidal alkaloids, excluding those found in *V. californicum*
